# Multidisciplinary Modalities Achieve Encouraging Long-Term Survival in Resectable Limited-Disease Esophageal Small Cell Carcinoma

**DOI:** 10.1371/journal.pone.0069259

**Published:** 2013-07-09

**Authors:** Xue Hou, Jin-Chang Wei, Jing-Xun Wu, Xin Wang, Jian-Hua Fu, Peng Lin, Hao-Xian Yang

**Affiliations:** 1 Department of Medical Oncology, Sun Yat-sen University Cancer Center, Guangzhou City, Guangdong Province, People’s Republic of China; 2 State Key Laboratory of Oncology in South China, Guangzhou City, Guangdong Province, People’s Republic of China; 3 Department of Thoracic Surgery, Linzhou Esophageal Cancer Hospital, Yaocun Town, Linzhou City, Henan Province, People’s Republic of China; 4 Department of Medical Oncology, the First Affiliated Hospital of Xiamen University; Xiamen City, Fujian Province, People’s Republic of China; 5 Department of Thoracic Surgery, Sun Yat-sen University Cancer Center,Guangzhou City, Guangdong Province, People’s Republic of China; University of Nebraska Medical Center, United States of America

## Abstract

**Background:**

The management of limited-disease esophageal small cell carcinoma is not well defined, and the role of surgery is still controversial. We aim to determine the optimal treatment strategy in limited-disease of esophageal small cell carcinoma.

**Methods and Findings:**

We conducted a retrospective review of 141 patients with limited-disease esophageal small cell carcinoma from 3 institutions in China who underwent treatment between July 1994 and September 2008, July 1994 and July 2011, and June 2004 and December 2010, respectively. The survival rate was calculated by the Kaplan-Meier method, and the log-rank test was used to assess the survival differences between the groups. Cox proportional hazards model were used to further determine the independent factors impacting overall survival. The median survival time was 16.1 months for the entire cohort of patients, with a 5-year survival rate of 6.7%. The median survival times for surgery alone, surgery combined with chemotherapy, surgery combined with radiotherapy, surgery combined with chemotherapy and radiotherapy, chemotherapy plus radiotherapy, and chemotherapy alone were 18.0 months, 15.0 months, 23.0 months, 25.0 months, 17.1 months, and 6.1 months, respectively; the corresponding 5-year survival rates were 0%, 15.4%, 0%, 38.9%, 0%, and 0%, respectively. For the 105 patients who underwent R0 resection, the median disease-free survival time was 12.0 months, with a 95% confidence interval of 9.5 months to 14.5 months. The multivariate Cox regression analysis demonstrated that advanced pathological staging (*p* = 0.003), and pure esophageal small cell carcinoma (*p* = 0.035) were independent factors decreasing overall survival.

**Conclusions:**

Our data suggested that multidisciplinary modalities achieved encouraging long-term survival in patients with resectable limited-disease of esophageal small cell carcinoma.

## Introduction

Esophageal small cell carcinoma (ESCC) is a rare and aggressive disease for which there is no recommended standard treatment for limited-disease ESCC patients at present [1]–[8]. Surgical resection, radiation therapy, and multi-agent chemotherapy have been used alone and in combination. However, due to the small number of cases reported in the literature, the management of ESCC is not well defined, and the role of surgery in limited-disease ESCC is still controversial [4], [6], [9]–[18].

In this study, we used data from multiple centers in China to retrospectively analyze the survival prognosis and risk factors of limited-disease ESCC, with the purpose of determining the role of surgery in ESCC. Using a large number of the multi-center database, we found that a multidisciplinary strategy that combined surgery with chemotherapy and radiotherapy is the way to achieve encouraging survival in patients with early stages of esophageal small cell carcinoma.

## Materials and Methods

### Ethics Statement

This study was approved by the Ethics Committee of the Sun Yat-sen University Cancer Center, Linzhou Esophageal Cancer Hospital, and the First Affiliated Hospital of Xiamen University. Since this is a non-randomized retrospective prognosis analysis and the data were de-identified and analyzed anonymously, the ethics committees waived the need for consent.

### Patient Selection

This is a multi-center retrospective study. We used data from the following 3 institutions: Sun Yat-sen University Cancer Center, Linzhou Esophageal Cancer Hospital, and the First Affiliated Hospital of Xiamen University. The data from these 3 centers were collected from the patients who were diagnosed between July 1994 and September 2008, July 1994 and July 2011, and June2004 and December 2010, respectively.

All of the patients included in the analysis were histologically defined as primary limited-disease ESCC, and the tumours were considered resectable before treatment. The ESCC was diagnosed using the World Health Organization (WHO)’s histological criteria [19].

The pretreatment workup for the patients included a physical examination, chest radiography, barium meal, B ultrasound or computed tomography (CT) scan of the abdomen, and gastroscopic examination. In all of the patients, a routine biochemical profile was also required. Unless clinically indicated, brain magnetic resonance imaging (MRI) and radioactive isotope bone scans were not routinely performed.

All patients were staged as limited disease according to the Veterans’ Administration Lung Study Group staging system [20]. Lesions confined to the esophagus and adjacent organs with or without regional lymph node involvement were classified as limited disease, whereas lesions spreading beyond the locoregional boundaries were classified as extensive disease. We excluded the cases with extensive disease. The limited diseases invading the main structures of the mediastinum and with bulky lymph nodes involvement which were considered unresectable were also excluded. The seventh edition of the American Joint Committee on Cancer (AJCC) staging system for esophageal cancer does not classify staging groups for ESCC [21]; however, it indicates that undifferentiated tumours of the esophagus should be stage-grouped in a manner that is similar to that of G3 squamous cell carcinoma [21]. Therefore, the staging classification (seventh edition) of the AJCC for esophageal squamous cell carcinoma was used for pathological staging of the cases underwent surgery [21].

### Surgical Procedure

For the patients who underwent surgical resection, the surgical procedures included primary tumour resection and lymph node dissection. The most commonly used surgical approaches included left thoracotomy, the Ivor-Lewis approach, and a cervico-thoraco-abdominal procedure. The left thoracotomy and the Ivor-Lewis procedure with anastomosis of the upper chest were mainly performed for tumours of the lower-third esophagus and some tumours of the middle-third esophagus. The cervico-thoraco-abdominal procedure was mainly used for tumours of the upper-third and middle-third esophagus. Extensive lymph node dissection in the posterior mediastinum and abdomen was systematically performed. Cervical lymphadenectomy was not systematically performed. For patients with cervical anastomosis, the lymph nodes exposed in the cervical incision were also dissected.

We defined the R0, R1, and R2 categories as resections with negative margins, microscopically positive margins, and grossly positive residual margins or M1 disease, respectively.

### Follow-up of Patients

In general, a follow-up examination was performed every 3 months for the first year, every 4 months for the second year, and twice a year thereafter. The routine examination during follow-up included a physical examination, blood chemistry, measurement of serum tumour markers, chest x-ray, esophagography, ultrasonography, and endoscopy. If the patient had specific symptoms, the examination was performed as soon as possible for a more careful assessment.

Operative death is defined as death within 30 days of operation or any time after operation if the patient did not leave the hospital alive. Cancer relapse included locoregional recurrence and distant metastasis.

March 2012 was the last censoring date for the survival analysis. The median time from the diagnosis to the last censoring date for the entire cohort was 77.4 months, ranging from 9.9 to 183.6 months.

### Statistical Analysis

Statistical analysis was performed using SPSS 16.0 (SPSS Inc, Chicago, Ill). The mean values were described as the mean ± standard deviation (SD). A Pearson Chi-square test was used to determine the significance of differences between groups for dichotomous variables. The survival rate was calculated by the Kaplan-Meier method, and the log-rank test was used to assess differences in survival between groups. The relationships between the survival and clinicopathologic variables were determined by Cox’s multivariate analyses. A 2-sided probability value of less than 0.05 was considered to be statistically significant. The survival time was measured from the date of treatment to the date of adverse event or last follow-up. Patients who were lost during the follow-up time period were censored at the last time of contact. Patients who were alive at the end of the study were also classified as censored for the purpose of data analysis.

## Results

### Patient Characteristics

ESCC was diagnosed in 1.8% (39 cases), 1.2% (61 cases), and 1.5% (10 cases) of all esophageal malignancies that underwent surgical resection during the time period at the Yat-sen University Cancer Center, Linzhou Esophageal Cancer Hospital, and the First Affiliated Hospital of Xiamen University, respectively. In total, 141 patients were diagnosed with ESCC, in which 110 patients underwent surgical resection from the 3 centers, and 31 patients received chemotherapy and/or radiotherapy from Sun Yat-sen University Cancer Center. All the cases were from Chinese Han population. The patients had a mean age of 57.5±8.6 years (median, 58 years; range, 38–76 years), with a male to female ratio of 3.1∶1. For the patients who did not receive surgery, all of them (31 cases) were diagnosed as ESCC by biopsy. For the patients that underwent surgical resection, 68 of them were diagnosed as ESCC by preoperative biopsy, while the other cases were diagnosed as poorly differentiated carcinoma (20 cases), squamous cell carcinoma (21 cases), and adenocarcinoma (1 case), respectively. In the patients that underwent surgery, pure ESCC accounts for 80.9% (89 cases) of patients diagnosed by the gross resection specimen, while the other cases were ESCC mixed with squamous cell carcinoma (18 cases, 16.4%) and mixed with adenocarcinoma (3 cases, 2.7%). There were 5 cases that underwent incomplete resection (R1 and R2). The general characteristics of the included patients are listed in Table 1. Among the 110 cases that underwent surgical resection combined with chemotherapy or/and radiotherapy, 2 of them (both from Sun Yat-sen University Cancer Center) were administered neoadjuvant chemotherapy plus surgery followed by adjuvant chemotherapy; the others underwent surgery plus postoperative chemotherapy or/and radiotherapy.

**Table 1:** Clinicopathological characteristics of the patients (n = 141).

		SYSUCC	LZECH	FAHXU	
Variable		No. (% of n)	No. (% of n)	No. (% of n)	*p*
Gender					
	Male	58 (82.9)	39 (63.9)	10 (100)	0.007
	Female	12 (17.1)	22 (36.1)	0 (0.0)	
Age (yrs)					
	<60	44 (62.9)	38 (62.3)	7 (70.0)	0.894
	≥60	26 (37.1)	23 (37.7)	3 (30.0)	
Tumor location					
	Upper third	8 (11.4)	6 (9.8)	0 (0)	0.306
	Middle third	41 (58.6)	44 (72.1)	6 (60.0)	
	Lower third	21 (30.0)	11 (18.0)	4 (40.0)	
Tumour length (cm)^a^					
	<5 cm	12 (30.8)	18 (29.5)	3 (0)	0.991
	≥5 cm	27 (69.2)	43 (70.5)	7 (70.0)	
AJCC stage					
	I	1 (1.4)	4 (6.6)	0 (0)	0.315
	II	27 (38.6)^ b^	18 (29.5)	2 (20.0)	
	III	42 (60.0)^ c^	39 (63.9)	8 (80.0)	
Pathology					
	Pure SC	64 (91.4)	48 (78.7)	8 (80.0)	0.111
	Mixed SC	6 (8.6)	13 (21.3)	2 (20.0)	
LV invasion^a^					
	Yes	29 (74.4)	46 (75.4)	4 (40.0)	
	No	10 (25.6)	15 (24.6)	6 (60.0)	
Treatment					
	S	19 (27.1)	20 (32.8)	3 (30.0)	<0.001
	S+C	17 (24.3)	27 (44.3)	3 (30.0)	
	S+R	1 (1.4)	8 (13.1)	0 (0)	
	S+C+R	2 (2.9)	6 (9.8)	4 (40.0)	
	C+R	20 (28.6)	-	-	
	C	11 (15.7)	-	-	
Surgical outcome^a^					
	R0	38 (97.4)	57 (93.4)	10 (100.0)	0.594
	R1	1 (2.6)	1 (1.6)	0 (0)	
	R2	0 (0)	3 (4.9)	0 (0)	

SYSUCC, Sun Yat-sen University Cancer Center; LZECH, Linzhou Esophageal Cancer Hospital; FAHXU, the First Affiliated Hospital of Xiamen University; AJCC, American Joint Committee on Cancer; SC, small cell; LV, lymphovascular; S, surgery; C, chemotherapy; R, radiotherapy. ^a^for the patients with surgical resection (n = 110); ^b^including 18 cases of pathological staging with surgical resection, 9 cases of clinical staging with non-surgical resection; ^c^ including 20 cases of pathological staging with surgical resection, 22 cases of clinical staging with non-surgical resection.

Seven cases combined with diabetes (5.0%), 9 (6.4%) cases combined with hypertension at the time of diagnosis. The co-morbidities were well controlled at the time of anti-cancer treatment, and no patient was rejected for surgery or chemo/radiotherapy because of the co-morbidities.

### Mortality and Morbidity

The operative mortality was 1.8% (2/110). The most common complications were pulmonary-cardiovascular disorders, laryngeal nerve palsy, and anastomotic leakage (Table 2). Of the 4 cases of anastomotic leakage, 1 died from pulmonary infection, and the other 3 cases were cured by persistent drainage and antibiotics. The other operative death was caused by acute respiratory distress syndrome. Both the operative deaths were without co-morbidities at the time of diagnosis. No chemo/radiotherapy related deaths occurred.

**Table 2:** Postoperative morbidity for 110 patients undergoing esophagectomy.

Complications	No. Patients (%)
Pneumonia	5 (4.5)
Pulmonary edema/acute respiratory distress syndrome	4 (3.6)
Ventricular tachycardia	12 (10.9)
Anastomotic leakage	4 (3.6)
Laryngeal nerve palsy	6 (5.5)
Chylothorax	2 (1.8)
Wound infection	1 (0.9)
Overall	34 (30.9)

### Relapse and Survival

Among the 11 cases received chemotherapy alone, 6 cases achieved complete responses and 5 cases achieved partial responses. Among the 20 cases received chemo/radiotherapy but not received surgical resection, 16 cases achieved complete responses and 4 cases achieved partial responses. Seventy-two cases were recorded with conclusive sites of tumour recurrence or distant metastases during the follow-up. The recorded recurrence or distant metastases sites were as follows: regional and distant lymph nodes (30 cases), liver (23 cases), lung (20 cases), pleura (4 cases), brain (5 cases), bone (6 cases), esophagogastric anastomotic stoma (2 cases), adrenal gland (1 case), and chest wall (1 case). The median survival times for patients with surgical resection and that without surgical resection were 18.6 months and 12.6 months, respectively; the corresponding 5-year survival rates were 8.4% and 0%, respectively (p = 0.111). The median survival times for surgery combined with chemotherapy and radiotherapy was longer than that of other groups (p = 0.018, Table 3). For the 105 patients who underwent R0 resection, the median disease-free survival was 12.0 months, with a 95% confidence interval of 9.5 months to 14.5 months.

**Table 3:** Overall survival for subgroups and the entire cohort of patients (n = 141).

				95% CI			
Variable		No.	Median survival (months)	Upper	Lower	5-year survival (%)	*p*
AJCC stage							
	I+II^a^	52	24.0	21.8	26.1	10.1	<0.001
	III	89	14.0	12.9	15.1	3.7	
Sex							
	Male	107	15.0	11.8	18.2	5.0	0.228
	Female	34	22.0	13.1	30.9	14.4	
Age (yrs)							
	<60	89	16.000	10.9	21.1	8.5	0.244
	≥60	52	17.100	13.5	20.7	0	
Tumor location							
	Upper third	14	14.0	10.3	17.7	0	0.961
	Middle third	91	16.1	11.4	20.8	6.9	
	Lower third	36	18.0	9.7	26.3	0	
Tumor length ^b^							
	<5 cm	33	25.0	19.6	30.4	13.1	0.010
	≥5 cm	77	15.0	13.4	16.6	5.8	
Pathology ^b^							
	Pure SC	89	15.0	11.6	18.4	9.7	0.048
	Mixed SC	21	25.0	19.9	30.1	10.5	
LV invasion ^b^							
	Yes	31	15.0	13.1	16.9	NA	0.189
	No	79	20.0	15.6	24.4	9.7	
Surgical outcome ^b^							
	Complete	105	18.0	14.0	22.0	6.3	0.476
	Incomplete	5	NA	NA	NA	5.3	
Treatment							
	S	42	18.0	11.6	24.4	0	0.018
	S+C	47	15.0	13.1	16.9	15.4	
	S+R	9	23.0	12.2	33.8	0	
	S+C+R	12	25.0	4.5	45.5	38.9	
	C+R	20	17.1	11.5	22.7	0	
	C	11	6.1	2.4	9.8	0	
Overall		141	16.1	12.1	20.1	6.7	NA

AJCC, American Joint Committee on Cancer; SC, small cell; LV, lymphovascular; S, surgery; C, chemotherapy; R, radiotherapy; NA, not available. ^a^only five cases were with stage I; ^b^pathological diagnosis by gross resection specimen for the patients with surgical resection (n = 110).

**Figure 1:**
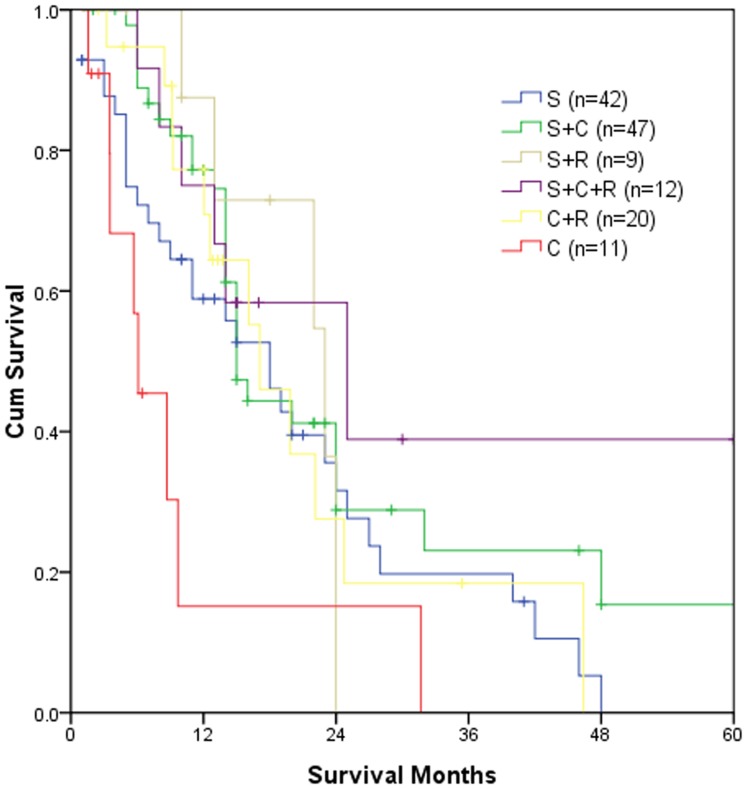
Survival curves for different treatment groups (n = 141). S, surgery; C, chemotherapy; R, radiotherapy. Among the six groups, *p* = 0.018; S vs. S+C, *p* = 0.155; S vs. S+R, *p* = 0.842; S vs. S+C+R, *p* = 0.155; S vs. C+R, *p* = 0.695; S vs. C, *p* = 0.064; S+C vs. S+R, *p* = 0.828; S+C vs. S+C+R, *p* = 0.483; S+C vs. C+R, *p* = 0.530; S+C vs. C, p<0.001; S+R vs. S+C+R, *p* = 0.434; S+R vs. C+R, *p* = 0.849; S+R vs. C, *p* = 0.061; S+C+R vs. C+R, *p* = 0.380; S+C+R vs. C, *p* = 0.010; C+R vs. CR, *p* = 0.013.

The overall survival data for the entire cohort of patients and subgroups are listed in Table 3. Figure 1 demonstrates the survival curves of different treatment groups.

The multivariate Cox regression analysis for the entire cohort of 141 patients demonstrated that advanced AJCC staging, and pure ESCC were independent factors decreasing overall survival (Table 4).

**Table 4:** Factors that affect the overall survival by multivariate Cox regression analysis for the entire cohort of patients (n = 141).

Variable		Relative risk	95% CI		*p*
			Upper	Lower	
Sex					
	Male	1			0.144
	Female	0.647	0.361	1.160	
Age (yrs)					
	<60	1			0.380
	≥60	1.233	0.772	1.970	
Tumour length					
	<5 cm	1			0.067
	Others a	1.734	0.962	3.126	
AJCC stage					
	I+II b	1			0.003
	III	2.080	1.292	3.349	
Tumor location					
	Upper third	1			0.765
	Middle third	0.813	0.401	1.649	
	Lower third	0.740	0.330	1.659	
Pathology					
	Pure SC	1			0.035
	Mixed SC	0.459	0.222	0.946	
LV invasion					
	No	1			0.865
	Yes	1.054	0.608	1.825	
	NA	0.877	0.467	1.649	
Treatment					
	S+C+R	1			0.587
	Others	1.272	0.534	3.032	

AJCC, American Joint Committee on Cancer; SC, small cell; LV, lymphovascular; NA, not available; S, surgery; C, chemotherapy; R, radiotherapy.

a including the tumours ≥5 cm in length and those not available for tumour length;

b only five cases were with stage I.

## Discussion

ESCC is a rare and highly aggressive malignancy, accounting for only 0.8% to 3.1% of all esophageal malignancies [1], [6]–[8], [10], [12]. In this study, the incidence was similar to that previously reported [1], [6]–[8], [10], [12]. The mean age of 57.5 years in this study was younger than the mean age of 63.8 years in a review of 199 cases [18], but was similar to that reported by Chinese colleagues [2], [9], [12], [13]. The ratio of male-to-female patients was 3.1∶1, which is higher than the ratio of 1.57∶1 in previous reports [18]. In most cases, the tumours were located in the middle third of the esophagus, compared to the lower third found in a previous review [18]. Our data suggest that there may be a difference between the general characteristics of ESCC patients from China and western countries.

Due to its rarity, the significance of surgical treatment in limited-disease ESCC is still controversial [10]. Some reports have emphasized that surgery should not be recommended because ESCC is a systemic disease [12], [15], [18]. However, other studies have suggested that surgery might have a potential impact on the long-term survival of patients with limited-disease ESCC [6], [9], [11], [13], [14], [16]. In view of the lack of specific prospective randomize controlled assays, classification into prognostic subgroups would likely aid in the selection of the best therapeutic approach to tackling this rare illness. In this study, we used data from multiple centers in China to retrospectively analyze the survival prognosis and risk factors of limited-disease ESCC. To our knowledge, this is the largest clinical report to date on the surgical outcome of ESCC. The large numbers utilized in this study permitted subgroup analyses. The median survival of 16.1 months for the entire cohort of patients in our data is encouraging for a disease as aggressive as ESCC, and it was much better than that found in a review of 199 cases from several published studies, which found a median survival of 8.5 months [18]. In our subgroup analysis, the patients with surgical resection showed a better 5-year survival rate than that without surgical resection (8.4% vs. 0%), suggesting that surgery may play an important role in appropriately selected limited-disease ESCC patients. Although the survival difference was not statistically significant, the fact that no one survived for 5 years in non-surgery group suggested that it was due to the small number of cases. Lv et al. reported on 126 ESCC patients, 85 of whom had limited disease [12], and showed a median survival of 14.0 months for limited disease [12], which was shorter than that of ours. Chen et al. recently reported 40 cases of limited-disease ESCC patients who underwent combined therapy, and the median survival in their study was 13.0 months [13]. It is interesting that in a multivariate Cox regression analysis, Chen et al.’s data suggested that operation was an independent factor favoring overall survival [13]. Sun et al. reported 73 cases of ESCC who underwent surgical treatment, and the 5-year survival rate was 8.2% [9], which is similar to that of our study. In Sun et al.’s study, a subgroup analysis of stage I -II cases showed that the 5-year survival rate was 18.2%; therefore, these authors suggested that surgery should be recommended for stage I–II ESCC patients. In a subgroup analysis, our data showed that surgery combined with chemotherapy and radiotherapy achieved the highest 5-year survival rate of 38.9%. Although the survival difference was not statistically significant when compared to chemotherapy plus radiotherapy group, the facts that no one survived for 5 years in non-surgery group and that there was only a 15.4% 5-year survival rate in the surgery combined with chemotherapy group indicated that surgery combined with chemotherapy and radiotherapy should be an appropriate first choice for resectable limited-disease ESCC.

Due to the systemic nature of ESCC, chemotherapy is recognized as necessary in its treatment, both for limited and extended disease. For locally advanced disease, radiotherapy should also be considered. Therefore, the controversy focuses on whether surgical resection is necessary in selected patients, and whether surgery or radiotherapy should be the first choice for localized therapy for limited-disease ESCC. However, the optimal radiation schema for ESCC has not been well established, and the current radiation strategy is adapted from small cell lung cancer (SCLC). Surgery as the primary modality of treatment for SCLC was initially abandoned after the results of the British Medical Council studies, in which surgery did not show a survival benefit compared with radiotherapy [22], [23]. However, in recent years, a general consensus has been achieved that patients with T1-2N0M0 SCLC should not be denied surgery [24], [25].This consensus indicates that the treatment strategy for limited-disease ESCC might also need to be adjusted. The histogenesis of ESCC may be APUD cells or multipotent reserve cells [26], [27], explaining the coexistence of small cell, squamous, and adenocarcinoma in the same lesions [28]. Our data showed that ESCC mixed with other pathological types accounted for 19.1% of the patients that underwent surgery. Previous studies have shown that the incidence rate of mixed differentiation was over 30% [18], [28]. However, the actual incidence rate of mixed pathological types may be higher than was reported if the entire specimen is analyzed. The other pathological types that are mixed in small cell carcinoma may cause resistance to radiotherapy, suggesting that surgical resection may be appropriate in selected patients for its potentially curative effect. In this study, the long-term survival of patients with mixed pathological types was better than that of patients with pure ESCC, further indicating that surgery should not be excluded from the multidisciplinary therapy of selected cases.

Our study has its limitations. The number in subgroups was small, especially for the chemo/radiotherapy subgroup and surgery combined with chemo/radiotherapy subgroup. The small numbers in these subgroups suggest that the results need further confirmation. In addition, the retrospective nature must be considered in interpreting our data.

In conclusion, our data suggested that multidisciplinary modalities achieved encouraging long-term survival in patients with resectable limited-disease of ESCC.
